# Diagnosis of epileptic seizure neurological condition using EEG signal: a multi-model algorithm

**DOI:** 10.3389/fmed.2025.1577474

**Published:** 2025-05-20

**Authors:** Mosleh Hmoud Al-Adhaileh, Sultan Ahmad, Alhasan A. Alharbi, Mohammed Alarfaj, Mukta Dhopeshwarkar, Theyazn H. H. Aldhyani

**Affiliations:** ^1^King Salman Center for Disability Research, Riyadh, Saudi Arabia; ^2^Deanship of E-Learning and Information Technology, King Faisal University, Al-Ahsa, Saudi Arabia; ^3^Department of Computer Science, College of Computer Engineering and Sciences, Prince Sattam bin Abdulaziz University, Al-Kharj, Saudi Arabia; ^4^Department of Computer Science, Dr. Babasaheb Ambedkar Marathwada University, Aurangabad, India; ^5^Department of Electrical Engineering, College of Engineering, King Faisal University, Al-Ahsa, Saudi Arabia; ^6^Applied College in Abqaiq, King Faisal University, Al-Ahsa, Saudi Arabia

**Keywords:** electroencephalography, EEG data classification, seizure detection, epilepsy, SMOTE

## Abstract

**Introduction:**

Affecting millions of individuals worldwide, epilepsy is a neurological condition marked by repeated convulsions. Monitoring brain activity and identifying seizures depends much on electroencephalography (EEG). An essential step that may help clinicians identify and treat epileptic seizures is the differentiation between epileptic and non-epileptic signals by use of epileptic seizure detection categorization.

**Methods:**

In this work, we investigated Machine learning algorithms including Random Forest, Gradient Boosting, and K-Nearest Neighbors, alongside advanced DL architectures such as Long Short-Term Memory networks and Long-term Recurrent Convolutional Networks for detecting epileptic seizures in terms of difficulties and procedures evolved depending on EEG data. The EEG data classification by applying ML and DL framework to improve the accuracy of seizure detection. The EEG dataset consisted of 102 patients (55 seizure and 47 non-seizure cases), and the data underwent comprehensive preprocessing, including noise removal, frequency band extraction, and data balancing using SMOTE to address class imbalance. Key features, including delta, theta, alpha, beta, and gamma bands, as well as spectral entropy, were extracted to aid in the classification process.

**Results:**

A comparative analysis was conducted, resulting in high classification accuracy, with the Random Forest model achieving the best results at 99.9% accuracy.

**Discussion:**

The study demonstrates the potential of EEG data for reliable seizure detection while emphasizing the need for further development of more practical and non-invasive monitoring systems for real-world applications.

## 1 Introduction

Epilepsy is a neurological condition that affects neurons in the brain. In many instances, epilepsy may not be curable, but it can be managed and controlled with proper care. This involves taking essential steps to ensure patients’ safety, especially in situations in which they might be driving, cooking, or simply being at home. With effective monitoring, patients can feel more confident in their daily activities, knowing that help is available when needed. This can minimize potential harm and reduce their dependence on others. This highlights the significance of proper management in epilepsy.

Epilepsy, a neurological condition, is recognized as a widespread issue that poses a significant risk to human life. Global statistics from the World Health Organization (WHO) indicate that around 50 million people worldwide are affected by epilepsy, establishing it as one of the most prevalent neurological diseases globally. Epilepsy affects individuals of all genders, including males and females, and it is also observed in children (1). Epilepsy refers to a neurological condition in which there are irregular disruptions in the usual functioning of the brain. These disruptions lead to seizures, which can differ in duration and effect from one individual to another. Seizures may be brief and go unnoticed or affect specific body parts or the entire body, occasionally resulting in unconsciousness.

Epilepsy can arise from acquired neurological insults (2) (e.g., oxygen deprivation, head trauma, and strokes) that damage brain tissue and disrupt normal electrical functioning. Genetic mutations affecting ion channels, neurotransmitters, and neural transmission can also predispose individuals to chronic seizures. Elucidating these precipitating factors enables better prevention and treatment of epilepsy. EEG is a non-invasive diagnostic tool that captures the electrical activity generated by brain neurons. Given the multi-channel signals from scalp electrodes and the necessity for long-term recordings, advanced signal processing methods have become indispensable for EEG-based detection (3).

A critical component of managing epilepsy is seizure detection, which involves categorizing EEG signals into seizure or non-seizure classes. This process is facilitated by identifying prominent features within the EEG signals. An important step in reducing the human and monetary costs of uncontrolled epilepsy is the development of methods for more precise seizure detection (4). According to Van de Vel et al. (5), beyond the pursuit of epilepsy treatment options, there is an increasing recognition of the need for effective epilepsy management strategies to enhance patient and caregiver quality of life. Non-EEG-based seizure detection technologies are receiving growing research attention due to their potential to improve care quality, peace of mind, and independence. A comprehensive literature review was carried out, and discussions were held with manufacturers of commercially available devices to gain further insights. The reported performance of non-EEG-based seizure detection devices showed a wide range of sensitivity, from as low as 2.2%–100%. In terms of false detections per hour, the range was 0–3.23 when compared with the gold standard of video-EEG. This underscores the varying reliability of these devices and the need for further research and development in this field.

EEG signals are prone to human error and are impractical for continuous monitoring. While automated systems leveraging machine learning and deep learning have shown promise, significant challenges hinder their widespread adoption in the real world.

Data Limitations EEG datasets often suffer from class imbalance, with far fewer seizure events than non-seizure data, leading models to overlook critical seizure patterns. Signal Complexity: EEG signals are inherently noisy, contaminated by artifacts from muscle movements, eye blinks, or environmental interference, complicating feature extraction. Computational Trade-offs: Deep Learning (DL) models (e.g., CNNs, LSTMs, transfer learning in DL, GRU, and transformers) excel at automatic feature learning but require substantial computational resources, making them unsuitable for low-power wearable devices (5). Conversely, traditional ML models, while efficient, rely on manual feature engineering, which risks missing subtle seizure signatures. Generalizability: Many algorithms perform well on controlled datasets but falter with patient-specific variability or ambulatory recordings.

This study aims to explore the potential of EEG data classification using machine learning techniques to enhance seizure detection. We conducted extensive preprocessing of the EEG data, including noise filtering, frequency band extraction, and data balancing, to ensure robust feature extraction and to improve model performance. By evaluating the effectiveness of different machine learning models, this work contributes to the growing body of research aimed at developing more accurate and efficient tools for epilepsy management. Furthermore, we emphasize the need for non-invasive, user-friendly monitoring systems that can complement EEG-based detection in real-world clinical applications. The main contributions of the article include a robust preprocessing pipeline combining noise filtering, frequency band extraction, and SMOTE-based class balancing, coupled with a comparative analysis of five models: Random Forest (RF), Gradient Boosting, KNN, LSTM, and LRCN. The RF classifier achieves state-of-the-art accuracy (99.9%). The paper is structured as follows: Section 2 reviews existing methodologies, Section 3 details the proposed framework, Section 4 presents empirical results and comparisons, and Section 5 concludes with clinical implications and future directions.

## 2 Literature review

Over 50 million individuals throughout the world are afflicted with epilepsy, a neurological disorder. Seizures that cannot be controlled occur repeatedly. To improve medical results and quality of life for epileptic patients, it is essential to monitor and diagnose seizures in a timely manner. Seizures may be quickly and accurately diagnosed using EEG data, which records the brain’s electrical activity. On the other hand, patients may find it obtrusive and complicated gear is usually required.

Recent years have seen tremendous growth in the area of epileptic seizure identification using EEG data, merit to the use of several ML and DL approaches. This literature review examines 23 studies that have contributed to this domain, categorizing them based on their methodological approaches, datasets used, and the specific aspects of seizure detection they address. The studies are grouped into four main categories: Traditional ML Approaches, DL Methods, Hybrid and Novel Approaches, and Comparative Studies and Reviews.

### 2.1 Traditional machine learning approach

Several studies have employed traditional ML techniques for seizure detection and classification, often focusing on feature extraction and selection methods. Fergus et al. (6) proposed a supervised ML method using the real dataset, achieving a sensitivity and specificity of 88%. This study demonstrated the potential of traditional ML methods in creating generalizable seizure detection models. Raghu et al. (7) presented a model that is computationally efficient by using a new feature known as a successive decomposition index. The system was evaluated using three different databases. Authors proposed support vector machine (SVM) classifiers, they achieved high sensitivity (95.80–97.53%) and low false detection rates (0.4–0.57/h) across all datasets. The use of multiple datasets in this study provided robust validation of their approach, highlighting the importance of diverse data in developing reliable seizure detection methods. Rani et al. (8) developed SVM approach for classifying a peak signal EEG signal. The system was used dataset that collected from Bonn University dataset. The SVM model achieved a remarkable 99.60% accuracy rate and a low error rate of 0.039. Almustafa (9) conducted a comprehensive comparison of various ML. These studies have demonstrated the continued relevance and effectiveness of traditional ML approaches in seizure detection, particularly when combined with innovative feature extraction methods. The high accuracies achieved by these methods suggest that they remain competitive with more complex DL approaches in certain scenarios.

### 2.2 Deep learning method

Due to automatically learn essential characteristics from raw EEG data, DL approaches have improved seizure detection accuracy and resilience. Liu et al. (10) created a hybrid bilinear DL network using CNNs and RNNs, model was scored 97.4% on the Temple University Hospital Seizure Corpus and 97.2% on EPILEPSIAE, demonstrating the power of neural network architectural composition. This research showed that CNNs, which excel in spatial feature extraction, and RNNs, which capture temporal relationships in EEG data, work well together.

The linear graph convolution network (LGCN) introduced by Zhao et al. (11) uses spatial interactions in EEG data using a Pearson correlation matrix to identify seizures. This novel method showed graph-based neural networks could capture intricate spatial correlations between EEG channels. Gabeff et al. (12) used the REPO2MSE cohort of scalp-EEG recordings from 568 epilepsy patients to construct a CNN-based model for online seizure identification. For clinical applications, online detection is key. This work addressed it. Chou et al. (13) tested four CNN architectures for video-EEG data analysis and found that their best model had 97.7% ictal stage accuracy. This work showed that CNNs can interpret multimodal data for seizure detection, indicating that adding visual information to EEG signals may improve detection. A 3D CNN-based automated epilepsy detection method by Sun and Chen (14) was very accurate. Their method used CNNs’ three-dimensionality to collect EEG signals’ spatial and temporal properties. This research proved the generalizability of their 3D-CNN-based technique by performing well across numerous datasets. Kunekar et al. (15) employed LSTM networks to identify seizures with 97% validation accuracy on the UCI-Epileptic Seizure Recognition dataset. It is observed that LSTM outperformed traditional algorithms in accuracy and precision. This work showed that RNNs can identify seizures by recording EEG data temporal dynamics. These DL methods demonstrate automated feature learning and complicated, high-dimensional EEG data processing. High accuracies across datasets show DL seizure detection technologies are getting more dependable.

### 2.3 Hybrid and novel approaches

Several studies have proposed innovative methods that combine different techniques or introduce novel concepts to improve seizure detection, often addressing specific challenges in the field or exploring unconventional approaches.

Bandarabadi et al. (16) presented a statistical methodology for selecting the preictal period, which serves as an indicator of seizure predictability. This study was used EGG recordings from 18 patients, provided insights into optimizing preictal periods for more precise classification models. This study contributed to the important area of seizure prediction, which has implications for early intervention and improved patient care.

Mert and Akan (3) introduced novel EEG analysis methodologies that achieved accuracy rates as high as 97.89%, demonstrating the potential of innovative signal-processing techniques in seizure detection. While the specific details of their approach were not provided in the summary, the high accuracy achieved suggests that there is still room for improvement in EEG signal analysis techniques.

Brari and Belghith (17) developed a machine learning framework leveraging chaos and fractal theories. Their approach, which included reconstructing EEG signals and extracting the Hurst fractal dimensions, achieved 100% accuracy on the Bonn EEG database using a small number of features and a linear classifier. This study highlighted the potential of applying concepts from complex systems theory to EEG analysis, offering a novel perspective on seizure detection.

Shah et al. (18) combined RNNs with a discrete wavelet transform for seizure detection. This hybrid approach demonstrated the benefits of combining wavelet-based feature extraction with the modeling capabilities of random neural networks.

Kantipudi et al. (19) presented an advanced complex Neural Network. This complex approach achieved an overall detection performance of 99.6% with a high F-measure (99%) and G-mean (98.9%). The study showed the potential of combining multiple advanced techniques, including bio-inspired optimization and specialized neural network architectures.

Ein Shoka et al. (20) introduced CNN model to classify EEG data using chaotic maps for addressing the crucial aspect of data privacy in medical applications while maintaining high classification performance. This study addressed the important issue of privacy preservation in medical data analysis, which is becoming increasingly relevant in the era of big data and interconnected healthcare systems.

Zeng et al. (21) applied a method that integrates deep and shallow learning techniques. The combined approach used a deep neural network for feature extraction, followed by PCA for dimensionality reduction and shallow classifiers for final classification, achieving nearly 100% accuracy on the Bonn dataset. This hybrid approach leveraged the strengths of both deep and traditional machine learning methods, demonstrating the potential benefits of such integrations.

These hybrid and novel approaches demonstrate the potential for significant improvements in seizure detection by combining different techniques or introducing innovative concepts. They often address specific challenges in the field, such as privacy preservation, computational efficiency, or the need for more interpretable models.

### 2.4 Comparative studies and reviews

Several studies have focused on comparing different methods or providing comprehensive reviews of the field, offering valuable insights into the relative performance of various approaches and highlighting areas for future research.

Bhandari et al. (22) introduced a comparative study in which seven raters reviewed EEG sharp. Their results showed that certain criteria in sensor space and source space analysis could achieve accuracy rates comparable to expert scoring, providing insights into the effectiveness of different EEG analysis methods. Singh and Kaur (23) designed a neural network classifiers and nonlinear EEG features, demonstrating high accuracy and AUC. Their study provided a comparison point for the effectiveness of nonlinear feature extraction in seizure detection and highlighted the importance of feature engineering in machine learning approaches.

Polat and Nour (24) proposed a hybrid method for seizure detection and classification and compared different SVM kernels and normalization techniques. Their study, which achieved accuracies of 76.70%–82.50%, showed the effects of preprocessing and classifier selection on detection performance. This study underscored the importance of careful parameter tuning and preprocessing in achieving optimal performance with traditional machine learning methods.

Farooq et al. (25) conducted a systematic literature review of ML techniques for seizure detection. Their review identified common feature extraction methods and classifiers, created a taxonomy of state-of-the-art solutions, and highlighted research gaps and challenges. This comprehensive review provided a valuable overview of the field, insights into trends, and directions for future research.

Hamlin et al. (26) explored the use of non-cerebral sensor data for seizure detection and compared the effectiveness of different sensor types and features. Their study, which achieved a mean ROC value of 0.9682, suggested the potential of multimodal approaches in improving seizure detection accuracy. This study opened up new possibilities for seizure detection by incorporating data from sensors beyond traditional EEG, potentially leading to more robust and versatile detection systems.

These comparative studies and reviews provide valuable insights into the relative performance of different methods and highlight areas for future research. They offer a broader perspective on the field and help researchers and practitioners understand the strengths and limitations of various approaches.

### 2.5 EEG datasets review

Epilepsy research and seizure detection have greatly benefited from the availability of diverse and comprehensive EEG datasets. This section provides review all type of datasets utilized in recent studies on epilepsy classification and seizure detection. These datasets vary in size, patient population, and recording methods.

#### 2.5.1 CHB-MIT dataset

The CHB-MIT dataset has been widely used in several studies for seizure detection and classification. Fergus et al. (6) employed this dataset in their supervised machine learning approach, achieving 88% sensitivity and specificity. Raghu et al. (7) utilized SVM classifiers on this dataset, resulting in 97.28% sensitivity and a false detection rate of 0.57/h. Zhao et al. (11) implemented a Linear Graph Convolution Network (LGCN) on the CHB-MIT data, achieving impressive results with 99.30% accuracy, 98.82% specificity, and 99.43% sensitivity. Shah et al. (18) combined Random Neural Networks (RNN) with Discrete Wavelet Transform (DWT) on this dataset, achieving 93.27% accuracy. Sun and Chen (14) also used this dataset in their 3D-CNN approach, reporting high accuracy, although the specific value was not provided in the summary.

#### 2.5.2 Bonn University dataset

The Bonn University dataset has been the foundation for several innovative approaches in seizure detection. Rani and Chellam (8) achieved a remarkable 99.60% accuracy using their Peak Signal Features (PSF) method combined with an SVM classifier on this dataset. Brari and Belghith (17) applied concepts from chaos and fractal theories to the Bonn dataset, achieving 100% accuracy. (18), in addition to their work on the CHB-MIT dataset, also used the Bonn dataset, achieving an even higher accuracy of 99.84% with their RNN and DWT combination. Zeng et al. (21) employed a hybrid approach combining deep and shallow learning techniques on this dataset, reporting nearly 100% accuracy.

#### 2.5.3 Temple University Hospital (TUH) dataset

The TUH dataset has been utilized in studies employing various ML and DL techniques. Liu et al. (10) achieved a 97.4% F1-score on this dataset using their hybrid bilinear DL network. Raghu et al. (7), as part of their multi-dataset study, applied SVM classifiers to the TUH data, achieving 95.80% sensitivity and a false detection rate of 0.49/h. Sun and Chen (14) included the TUH dataset in their 3D-CNN study, reporting high accuracy, although the specific value for this dataset was not provided in the summary.

#### 2.5.4 EPILEPSIAE dataset

The EPILEPSIAE dataset was used by Liu et al. (10) in their comprehensive study employing a hybrid bilinear deep learning network. On this dataset, their approach achieved a 97.2% F1-score, demonstrating the effectiveness of their method across different datasets.

#### 2.5.5 UCI-epileptic seizure recognition dataset

Kunekar et al. (15) utilized the UCI-Epileptic Seizure Recognition dataset in their study focusing on LSTM networks for seizure detection. Their approach achieved a validation accuracy of 97% on this dataset, highlighting the potential of recurrent neural networks in capturing the temporal dynamics of EEG signals for seizure detection.

#### 2.5.6 REPO2MSE dataset

Gabeff et al. (12) used the REPO2MSE dataset, which consists of scalp-EEG recordings from 568 epilepsy patients, to develop their CNN-based model for online epileptic seizure detection. Table 1 given highlight the importance of standardized, publicly available datasets in advancing seizure detection research.

**Table 1:** Summary of EEG datasets.

Studies	Dataset	Description
Fergus et al. (6), Raghu et al. (7), Zhao et al. (11), Sun and Chen (14), and Shah et al. (18)	CHB-MIT	Scalp EEG data from 23 pediatric subjects with intractable seizures, recorded at the Children’s Hospital Boston. Contains 686 h of EEG recordings.
Rani et al. (8), Brari and Belghith (17), Shah et al. (18), and Zeng et al. (21)	Bonn University	Consists of 5 subsets (Z, O, N, F, S) each containing 100 single-channel EEG segments of 23.6-s duration. Sets Z and O are from healthy subjects, N and F from seizure-free intervals, and S contains seizure activity.
Raghu et al. (7), Liu et al. (10), and Sun and Chen (14)	Temple University Hospital (TUH)	Large-scale dataset of clinical EEG recordings from Temple University Hospital. Contains over 30,000 EEG records from more than 16,000 patients.
Liu et al. (10)	EPILEPSIAE	European database of long-term EEG data from epilepsy patients. Contains both scalp and intracranial EEG recordings.
Kunekar et al. (15)	UCI-Epileptic Seizure Recognition	Dataset from UCI Machine Learning Repository, containing 11,500 EEG recordings, each 1 s long, classified into 5 categories.
Gabeff et al. (12)	REPO2MSE	Cohort of scalp-EEG recordings from 568 epilepsy patients. Specific details not provided in the summary.

### 2.6 Conclusion of the EEG section review

The reviewed studies demonstrate significant progress in seizure classification and detection based on EEG signals. Traditional machine learning approaches continue to show effectiveness, particularly when combined with innovative feature extraction methods. The studies of Fergus et al. (6), Raghu et al. (7), and Rani and Chellam (8) show the potential of these methods when applied with careful feature engineering and selection.

Deep learning techniques, especially CNNs and LSTMs, have demonstrated remarkable performance in automatically learning relevant features from raw EEG data. Liu et al. (10), Zhao et al. (11), and Sun and Chen (14) revealed the power of these approaches in capturing complex spatial and temporal patterns in EEG signals. The high accuracies achieved by these methods across various datasets suggest that they are becoming increasingly reliable for seizure detection tasks.

Hybrid and novel approaches, such as those leveraging Brari and Belghith’s chaos theory (17), fractal dimensions, and Zhao et al. (11) graph neural networks have shown promise in improving detection accuracy and addressing specific challenges in the field. These innovative methods often combine the strengths of different approaches or introduce new concepts from other domains, pushing the boundaries of what is possible in seizure detection.

The integration of multiple data sources and sensor types, as seen in Hamlin et al.’s study (26), suggests promising directions for more robust seizure detection systems. This multimodal approach could lead to detection systems that are less prone to false positives and more adaptable to different patient populations.

Comparative studies and reviews, such as those by Kural et al. (22) and Farooq et al. (25), provide valuable insights into the relative performance of different methods and highlight areas for future research. These studies help contextualize individual research efforts within the broader landscape of seizure detection techniques.

However, challenges remain in terms of generalizability across different datasets and patient populations, as well as in reducing false-positive rates and detection delays. The need for larger, more diverse datasets and standardized evaluation metrics is evident from the literature. Many studies use different datasets and evaluation metrics, making direct comparisons challenging. Table 2 reviews studies on EEG-based seizure detection by summarizing the methodologies, technologies, and results of various research efforts and focusing on the effectiveness and accuracy of EEG applications in detecting seizures.

**Table 2:** A review of studies of EEG-based seizure detection.

Study	Data	Preprocessing	Models/Algorithms	Results
Liu et al. (10)	Temple University, EPILEPSIAE dataset	exploit the frequency (STFT), analysis data	Hybrid bilinear deep learning network (CNNs + RNNs)	F1-score: 97.4%
Fergus et al. (6)	CHB-MIT dataset	Simple, filter, features extraction	k-NN, SVM, NN, DT	Sensitivity 88%, AUC: 93%
Mert and Akan (3)	Various EEG recordings	Digitalize, filter, Normalize frequency	Novel EEG analysis methods	Accuracy: 97.89%
Raghu et al. (7)	Ramaiah Medical College, CHB-MIT	Feature extraction (SDI)	SVM	Sensitivity: 97.53%
Bhandari et al. (22)	1,001 patients (video-EEG) EMG Data	Record, sample and filter the data	Analysis of EEG sharp transients	92% Accuracy
Zhao et al. (11)	CHB-MIT dataset	Pearson correlation matrix	Linear Graph Convolution Network (LGCN)	Accuracy: 99.30%, Sensitivity: 99.43%
Rani et al. (8)	Bonn University dataset	Peak Signal Features (PSF)	SVM, DT, KNN	Accuracy up to 99.60% with SVM
Aayesha et al. (28)	Bonn and CHB-MIT datasets	Feature extraction	KNN, FRNN	Accuracy: up to 99.81%
Gabeff et al. (12)	REPO2MSE cohort	Simple, segment and split the data	CNN	F1-score: 0.873, 90% seizure detection
Brari and Belghith(17)	Bonn EEG database	EEG signal reconstruction	Chaos and fractal theories	Accuracy: 100%
Chou et al. (13)	Video-EEG data	Not specified	Four CNN architectures	97.7% accuracy for ictal stage
Shah et al. (18)	CHB-MIT, BONN datasets	DWT	RNN, ANN, SVM	CHB-MIT: 93.27%, BONN: 99.84%
Polat and Nour (24)	Not specified	Z-score, Minimum-Maximum, MAD normalizations	SVM (Linear, Cubic, Medium Gaussian)	76.70–82.50%
Kantipudi et al. (19)	Not specified	FLHF	GBSO, TAENN	99.6%, F-measure: 99%, G-mean: 98.9%
Almustafa (9)	Not specified	Not specified	Random Forest, K-NN, Naïve Bayes, Logistic Regression, DT, Random Tree, J48, SGD	97% accuracy,
Kunekar et al. (15)	UCI-Epileptic Seizure Recognition dataset	Not specified	LSTM, Logistic Regression, SVM, KNN, ANN	97% Accuracy
Hamlin et al. (26)	Data from 15 patients	LDA	Not specified	Mean ROC: %96.8
Zeng et al. (21)	Bonn dataset	PCA	CNN, shallow classifiers	~100% Accuracy
George et al. (29)	KITS, TUH databases	TQWT, entropies	PSO, ANN	KITS: 100%, TUH: 88.8–97.4% Accuracy

**Figure 1:**
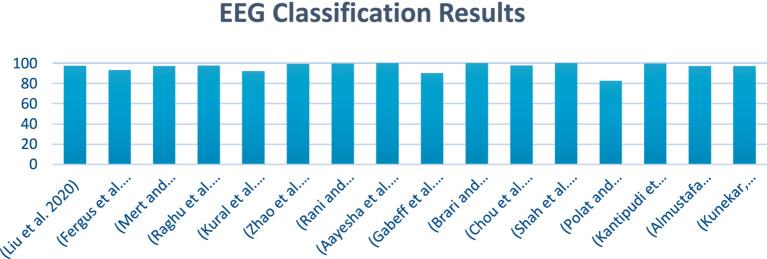
EEG classification result.

Figure 1 illustrates a summary of the EEG classification results. It provides a visual representation of how different EEG signals have been classified and shows the accuracy and performance of the classification model. It presents the various metrics and comparisons, helping to understand the effectiveness of the approach used to distinguish between different brain wave patterns.

## 3 Methodology

**Figure 2:**
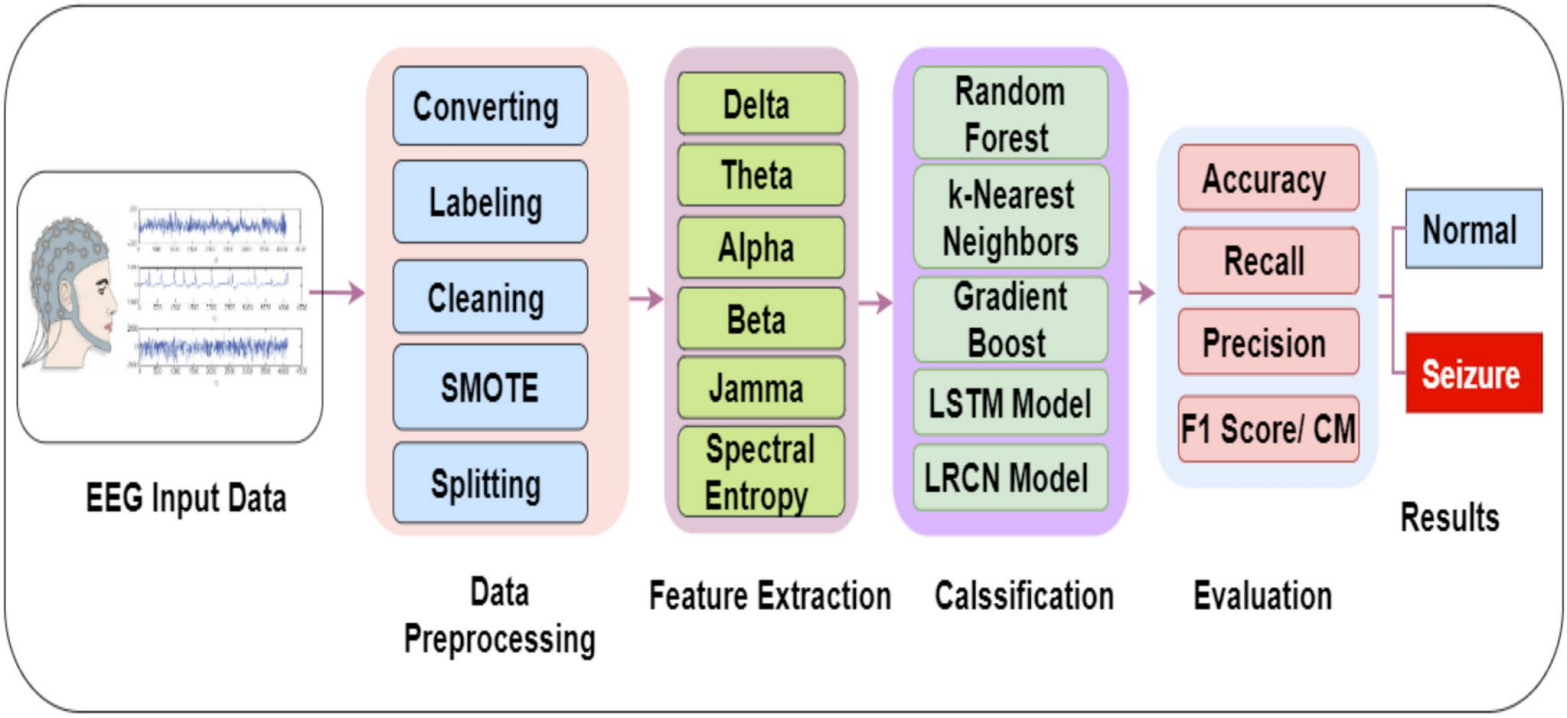
Proposed methodology for EEG data classification and seizure detection.

The proposed system is being investigated using a real EGG dataset. Various algorithms were employed to enhance the existing methods for modeling and detecting seizure diseases. This research presents a detailed overview of the training and validation methodologies employed for the RF, GB, LSTM, and LRCN models. The outlined method structures the approach employed to identify seizures through EEG data, as illustrated in Figure 2.

### 3.1 EEG dataset acquisition

EEG data were collected from a group of patients who had continuous video-EEG monitoring for an extended duration at two medical institutions in Denmark: Aarhus University Hospital and the Danish Epilepsy Center in Dianalund (22). The data collection period was from January 2012 to September 2017. During the diagnostic evaluation phase, sharp transients were initially identified and marked. Subsequently, two authors conducted a comprehensive review of these marked transients. Through collaborative analysis, a consensus was established among the experts, confirming the initial marking as a sharp transient, regardless of its manifestation of epileptiform characteristics. This selected sharp transient was then subjected to further evaluation to ensure compliance with the predetermined selection criterion. In the dataset, there were 100 files in the European Data Format (EDF), comprising data from 55 epileptic patients and 47 non-epileptic patients of different ages and genders. On December 18, 2017, the dataset that was used for this research was recorded. A sample rate of 500 Hz was used to get the EEG data, since this is the industry standard for collecting the important frequency content in EEG signals. The raw data was further processed using a 250 Hz low-pass filter. The EEG recording system employed in this study comprised 26 channels, enabling the simultaneous measurement of brain activity from multiple scalp locations. Table 3 outlines the EEG dataset content and features, such as the number of patients and class.

**Table 3:** EEG dataset content.

Class	Number of patients
Normal	55
Seizure	47

### 3.2 Preprocessing

**Figure 3:**
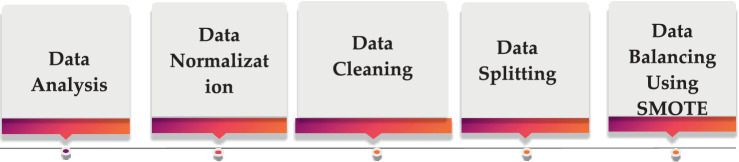
EEG data preprocessing steps.

In the data preprocessing phase, the raw EEG data undergo filtering to extract the relevant frequency bands of interest. Specifically, the following frequency bands are extracted: alpha (8–12 Hz), beta (13–30 Hz), theta (4–7 Hz), and gamma (above 30 Hz). These frequency bands are commonly analyzed in EEG studies because of their associations with various cognitive and physiological processes. It is crucial to preprocess the EEG data appropriately to ensure the reliability and validity of subsequent analyses (27). The filtering step is essential for isolating the frequency bands of interest and minimizing the influence of irrelevant signal components or noise. The extraction of these specific frequency bands facilitates the investigation of their potential correlations with the cognitive or physiological processes under study, as shown in Figure 3.

#### 3.2.1 Data labeling

In this process, we labelled all of the EEG recordings in the dataset according to the patient’s status. We used the numbers “1” to denote normal EEG data and the number “0” to denote seizures. While training, the classification algorithm benefits from this labeling as it allows it to differentiate between the two groups.

#### 3.2.2 Data normalization

The EEG characteristics were on the same scale, we normalized the data. If you want to make sure that the learning process is not overloaded with features with out-of-range values, normalization is a must. Z-score normalization method was used for scaling the rows of EGG dataset.

#### 3.2.3 Data cleaning

Initial data cleaning was performed to address any missing values within the features. The mean imputation technique was utilized, where missing values in any given feature were replaced with the mean value of that feature. This method was implemented using the SimpleImputer class from the sklearn.impute module, configured with strategy = ‘mean’. The transformation was applied to all feature columns, excluding the ‘label’ column, which represents the target variable.

#### 3.2.4 Data balancing using SMOTE

**Figure 4:**
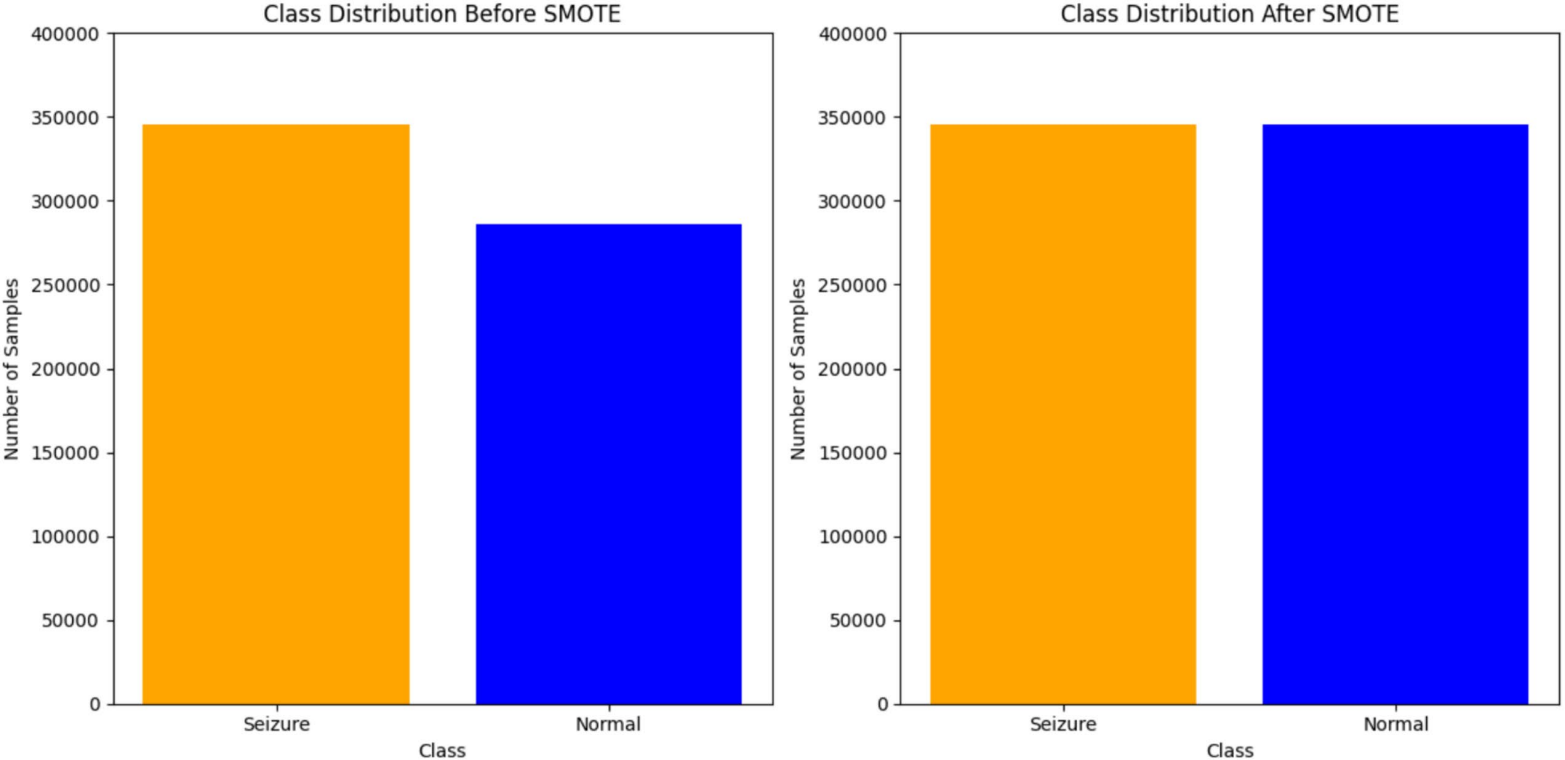
EEG data distribution before and after applying SMOTE.

SMOTE technique used to address class imbalances in datasets. One step in processing SMOTE data is to use synthetic samples for the minority class. This ensures that the distribution of classes is balanced. The algorithm works by identifying the KNN for each minority class sample and creating new synthetic samples along the line segments that join the minority class sample and its neighbors. The synthetic samples are generated by randomly selecting one of the KNN and introducing a perturbation along the line segment joining the two samples. This approach was implemented using the SMOTE class from the imblearn. over_sampling library with a random_state set for the reproducibility of results. The resampling process adjusted the dataset to ensure an equal representation of both classes, mitigating the potential effect of class imbalance on the subsequent analysis and modeling steps. Figure 4 illustrates the distribution of EEG data before and after applying SMOTE.

#### 3.2.5 Data splitting

Two subsets, training and testing, were taken from the dataset. A data allocation of 80% for training and 20% for testing the machine learning model is known as an 80/20 split. By splitting the data in this way, we can train the model on one set of data and then evaluate it on another set, which stops overfitting and lets the model generalize.

#### 3.2.6 Heatmap of amplitude differences

**Figure 5:**
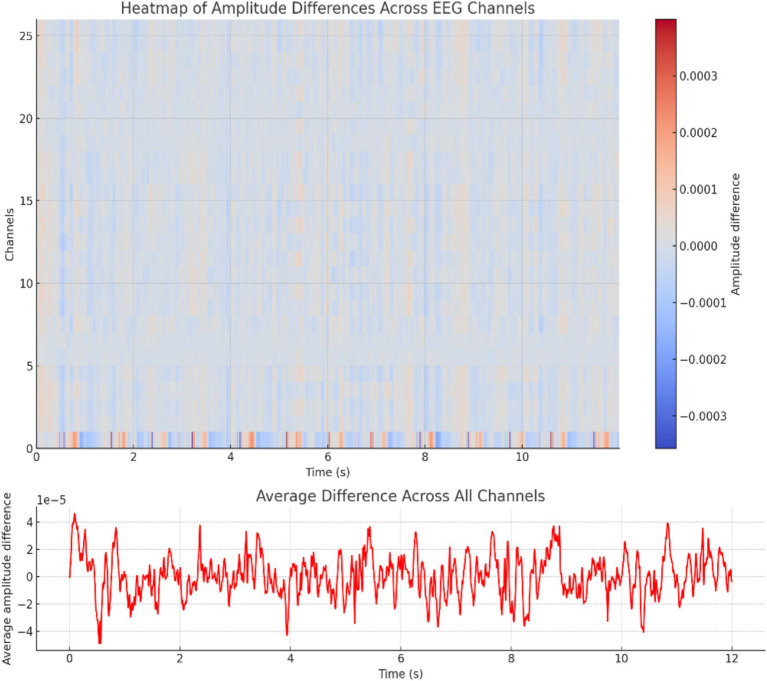
Heat map of amplitude differences.

The profound complexities underlying epileptic seizures necessitate a multifaceted approach to elucidate their intricate mechanisms. The study presents a comprehensive spatiotemporal analysis of EEG data, leveraging the visual potency of heat maps to delineate amplitude variations across cortical regions. By comparing seizure and non-seizure conditions, the proposed methodology quantifies the dynamic shifts in neural activity, transitioning seamlessly from negative to positive amplitude deviations through a “coolwarm” color palette. This graphical representation not only facilitates the localization of epileptogenic foci but also elucidates the propagation patterns of seizure activity, thereby contributing to a holistic understanding of the pathophysiological processes underlying this neurological disorder. As shown in Figure 5, the knowledge acquired from this study has great consequences for the formulation of focused treatment strategies and the progress of our understanding of the complex neural dynamics controlling seizure events.

#### 3.2.7 Spectral analysis

**Figure 6:**
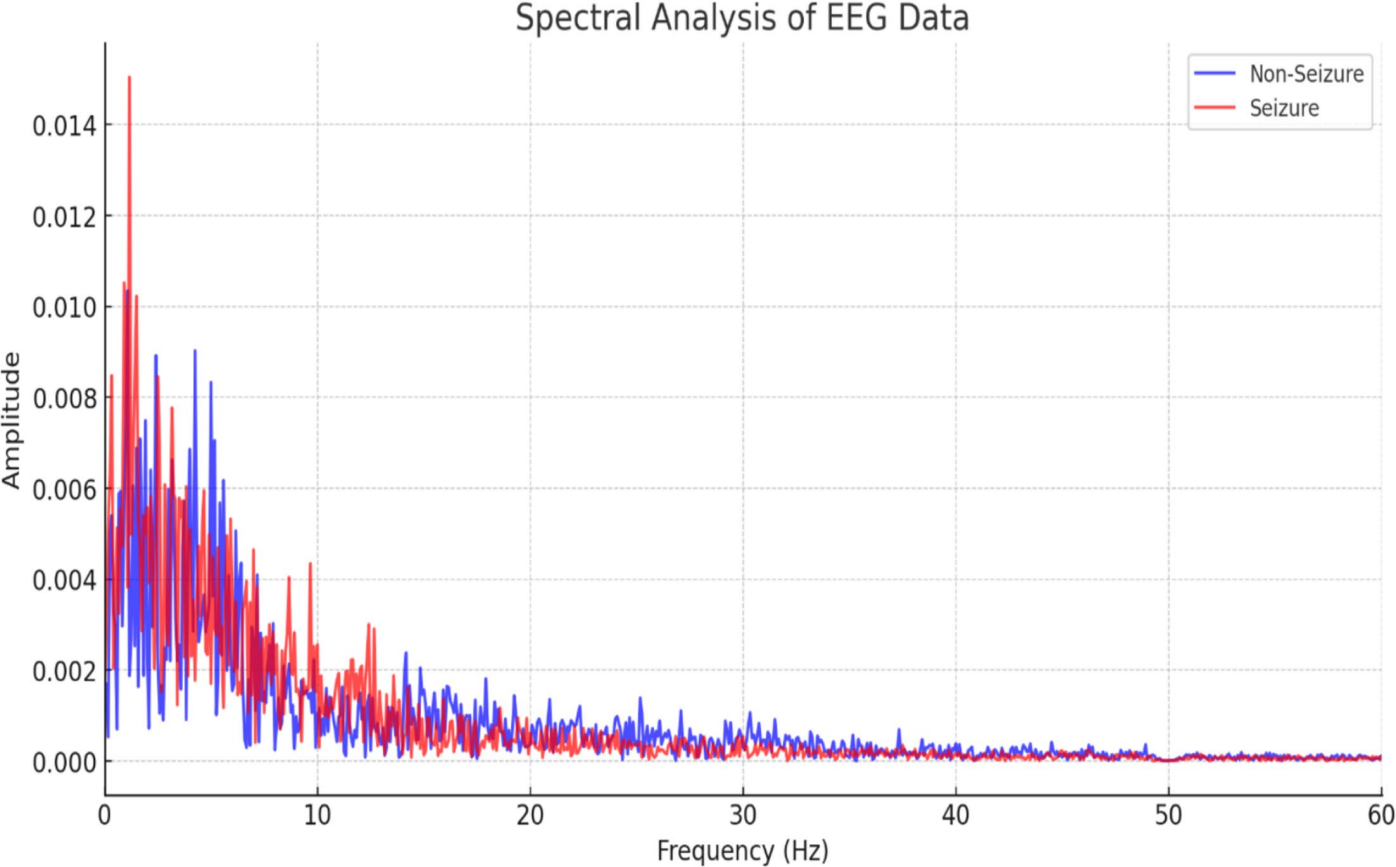
Spectral analysis of EEG signals of seizure and non-seizure cases.

This study used Fourier spectral analysis of EEG data to elucidate the frequency domain signatures that differentiate seizure and non-seizure neural dynamics in epilepsy. The spectral power distributions derived from these analyses revealed pronounced amplitudes across specific frequency bands during seizure activity, which is indicative of heightened neuronal synchronization. By contrast, the non-seizure condition exhibited reduced spectral power, reflecting normal neural oscillations. By characterizing these distinct frequency profiles, this work sheds light on the neurophysiological underpinnings of epileptic seizures and pathological hypersynchrony and paves the way for improved therapeutic interventions, as shown in Figure 6.

### 3.3 Feature extraction

**Figure 7:**
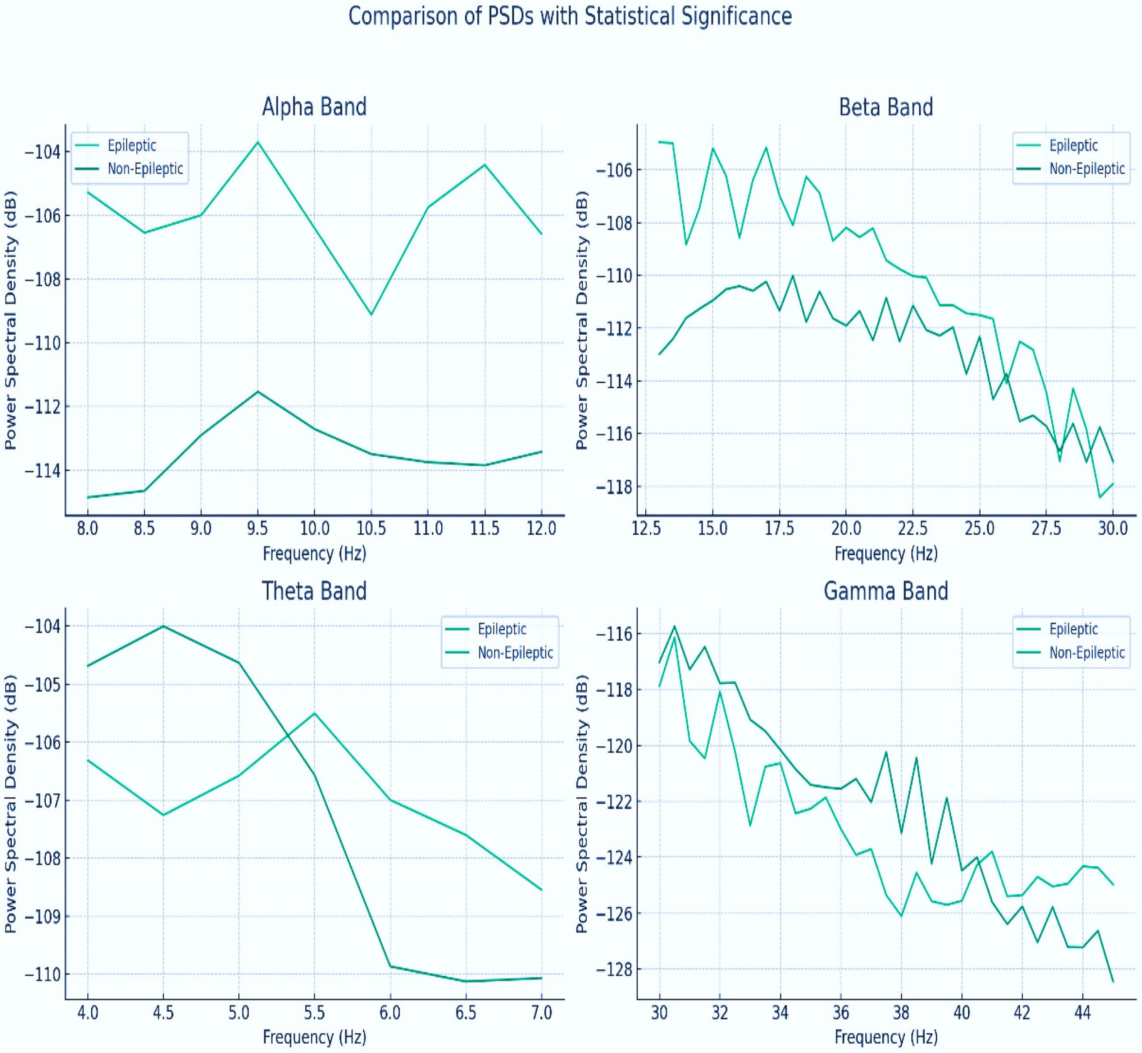
PSD for the alpha, beta, theta, and gamma bands between epileptic and non-epileptic patients.

This study analyzed the power spectral density (PSD) levels across different frequency bands to investigate the differences in neural activity between epileptic and non-epileptic patients. The epileptic patient exhibited distinct PSD levels compared with the non-epileptic patient, suggesting variations in their underlying neural activity patterns. The frequencies at which the difference in PSD between the two patients was statistically significant (*p* < 0.05) were identified, indicating that the observed differences in brain activity were unlikely due to chance. Significant differences at certain frequencies, such as increased power in the theta and gamma bands, could reveal specific brain activity patterns associated with epilepsy, including the presence of epileptic networks outside of seizure events. These findings contribute to a better understanding of the neurophysiological underpinnings of epilepsy and hold promise for improving diagnostic and monitoring techniques and for guiding more targeted interventions for the management of epilepsy, as shown in Figure 7.

In this section, several features were extracted from the EEG signals to enable the classification of epileptic and non-epileptic patients. These features capture different aspects of neural activity and provide valuable information for distinguishing between the two groups. The extracted features are as follows:

- *Delta*

Usually covering 0.5 to 4 Hz, this function shows the PSD in the delta frequency region. Deep sleep phases are linked to delta waves, which are also well-known to be involved in many cognitive functions like memory and attention.

- *Theta*

The theta feature corresponds to the PSD in the theta frequency band, which ranges from 4 to 8 Hz. Theta oscillations are linked to cognitive processes such as memory formation, spatial navigation, and emotional regulation.

- *Alpha*

The alpha feature is derived from the PSD in the alpha frequency band, typically between 8 and 12 Hz. Alpha waves are prominent during relaxed wakefulness and are believed to play a role in attention and information processing.

- *Beta*

This feature represents the PSD in the beta frequency band, ranging from 13 to 30 Hz.

- *Gamma*

The gamma feature corresponds to the PSD in the gamma frequency band, which encompasses frequencies above 30 Hz. Gamma oscillations are involved in various cognitive functions, including perception, attention, and memory.

- *Spectral entropy*

A estimate of the complexity or irregularity of the EEG signal, spectral entropy It may help to identify aberrant patterns of brain activity by providing details on the distribution of power across many frequency ranges.

The power spectral density (PSD) of gamma band (30 + Hz) emerged as the most discriminative feature, showing statistically significant amplitude increases during seizures (*p* < 0.05, Figure 7). This aligns with neurophysiological evidence linking high-frequency oscillations to epileptic hyperexcitability. The theta band (4–8 Hz) also demonstrated utility, though with marginally lower significance. Other bands (delta, alpha, and beta) contributed minimally, as their PSD distributions overlapped between seizure and non-seizure states.

Spectral entropy, quantifying signal irregularity, effectively captured abrupt changes in EEG complexity during seizures. It achieved a feature importance score of 0.180.18 in the Random Forest (RF) model, complementing gamma band analysis to reduce false positives caused by non-stationary noise.

Commonly utilized in EEG analysis, these characteristics have been shown to be useful in distinguishing and defining many brain states and disorders, including epilepsy. Table 4 summarizes the obtained characteristics; they will be input for categorization techniques.

**Table 4:** EEG extracted features summary.

Feature	Description
Delta	PSD in the delta frequency band (0.5–4 Hz)
Theta	PSD in the theta frequency band (4–8 Hz)
Alpha	PSD in the alpha frequency band (8–12 Hz)
Beta	PSD in the beta frequency band (13–30 Hz)
Gamma	PSD in the gamma frequency band (above 30 Hz)
Spectral entropy	Measure of the complexity or irregularity of the EEG signal

### 3.4 Modeling

In the classification stage, the EEG data was analyzed using four models: RF, GB, KNN, LSTM, and LRCN. The RF constructs multiple decision trees and uses majority voting for classification, well-suited for high-dimensional, nonlinear data like EEG signals. Gradient Boosting iteratively combines weak models to capture complex patterns. LSTM, a recurrent neural network variant, can learn long-term dependencies in sequential data such as EEG for identifying seizure patterns. LRCN combines convolutional layers for spatial feature extraction with LSTM for temporal modeling, making it effective for seizure detection and classification from EEG recordings. The specific architectures of these diverse machine learning and deep learning models were previously detailed, Table 5 lists EEG classification models. Justifications for each model in the context of EMG data classification between normal and seizure cases:

**Table 5:** EEG classification models.

No	Model
1	Random Forest Model
2	Gradient Boost Model
3	K-Nearest Neighbors Model
4	LSTM Model
5	LRCN Model

#### 3.4.1 Random Forest model

Random Forest Classifier excels in handling complex EMG data due to its ensemble nature. Combining many decision trees, each tuned on random selections of data and attributes, helps to detect complex trends in muscle activity signals. This approach is particularly effective for seizure detection, as it can identify subtle differences in EMG characteristics. The model’s feature importance ranking also provides insights into which aspects of the EMG signal are most predictive of seizures, aiding in both classification and physiological understanding.

#### 3.4.2 Gradient boost model

Gradient Boosting is well-suited for EMG classification due to its sequential learning process. Approaches the building of a series of weak learners, generally decision trees, in a stage-by-stage manner, with the main aim of fixing errors generated by previous models. This approach allows it to capture fine-grained differences in EMG patterns between normal and seizure states. Gradient Boosting’s ability to handle non-linear relationships and its robustness to outliers make it effective in dealing with the variability often present in EMG data during seizures.

#### 3.4.3 K-nearest neighbors model

The K-Nearest Neighbors model is valuable for EMG classification due to its non-parametric nature. It does not assume any specific distribution of the data, making it adaptable to the complex and often non-linear patterns in EMG signals during seizures. By classifying based on the majority class of nearby data points in the feature space, KNN can effectively capture local patterns in muscle activity. This local decision-making is particularly useful for identifying seizure-related EMG characteristics that may vary across patients or types of seizures.

#### 3.4.4 LSTM model

Long Short-Term Memory networks can process sequential data and record long-term dependencies, they are especially appropriate for EMG data interpretation. EMG signals during seizures often exhibit temporal patterns that evolve over time. LSTM’s gating mechanism allows it to selectively remember or forget information, making it adept at identifying relevant temporal features in the EMG signal that distinguish seizure activity from normal muscle function. This temporal modeling capability is crucial for detecting the onset and progression of seizures in EMG data.

#### 3.4.5 LRCN model

The LRCN combines the strengths of both CNNs and LSTMs, making it highly effective for EMG-based seizure detection. The CNN component excels at extracting spatial features from the EMG signal, potentially identifying characteristic frequency patterns or signal morphologies associated with seizures. The LSTM layer then processes these features sequentially, capturing the temporal evolution of muscle activity during seizure events. This dual approach allows LRCN to simultaneously analyze both the spatial and temporal aspects of EMG data, potentially leading to more accurate and robust seizure detection.

## 4 Results and discussion

In this subsection, we explore the performance of EEG classification for seizure detection using four models: GB, RF, K-NN, LSTM, and LRCN. The objective was to assess and compare their effectiveness in identifying seizures from EEG data. The results are detailed in the accompanying tables and figures, which present the potential of these models in advancing neurological diagnostics. Table 5 outlines the EEG classification models.

### 4.1 Evaluation matrix

The ML and DL model were evaluated by using evaluation matrix. The Equations 1–5 of evaluation metrics can be defined as follows:


(1) $$\text{Accuracy}=\frac{\mathrm{TP}+\mathrm{TN}}{\mathrm{FP}+\mathrm{FN}+\mathrm{TP}+\mathrm{TN}}\times 10$$

(2) $$\text{Sensitivity}\;\frac{\text{True Positives}\;}{\text{True Positives}+\text{False positives}\;}$$

(3) $$\text{Recall}=\frac{\mathrm{TP}}{\mathrm{TP}+\mathrm{FN}}$$

(4) $$f1-\text{score}=2*\frac{\text{precision}\times \text{Sensitivity}}{\text{precision}+\text{Sensitivity}}*100\;$$

(5) $$\text{Precision}=\frac{\mathrm{TP}}{\mathrm{TP}+\mathrm{FP}}$$

### 4.2 Environment setup

All experiments were conducted on a laptop with the following specifications: Intel Core i7 processor, 16GB RAM, and an NVIDIA GeForce RTX 3070 GPU with 8GB VRAM. The software environment consisted of Python 3.9 running within Anaconda, with TensorFlow version 10.1.2 employed for deep learning tasks.

### 4.3 Results of the GB model

**Figure 8:**
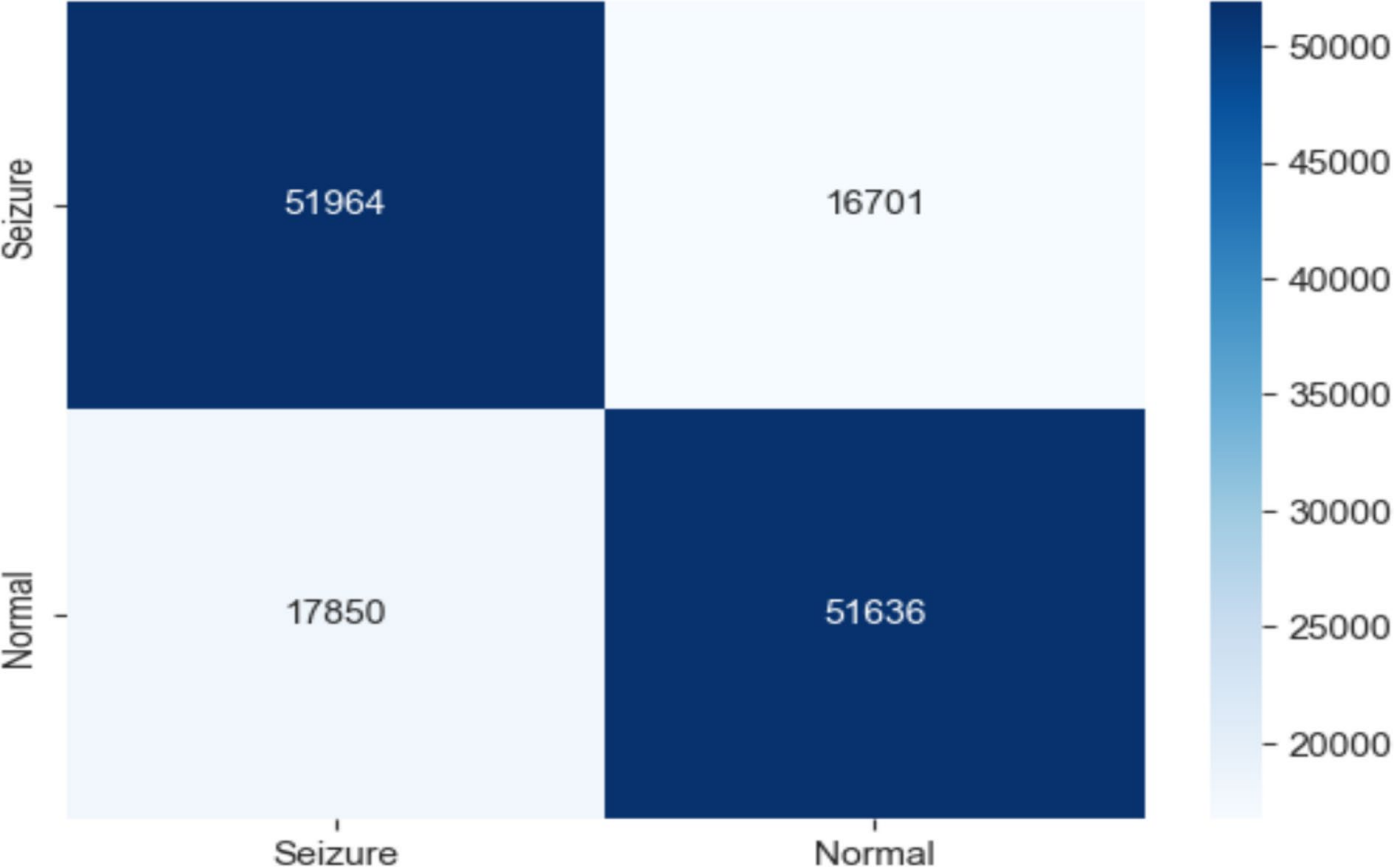
Confusion matrix of EEG data using the GB model.

This work classified epileptic and non-epileptic patients using Gradient Boosting (GB) model depending on EEG features. With an accuracy of 0.750, a precision of 0.756, a recall of 0.743, an F1 score of 0.749, and a ROC AUC score of 0.835 the model was able to differentiate between the two groups. With 51,964 true negatives, 51,636 true positives, 16,701 false positives, and 17,850 false negatives, the confusion matrix as shown in Figure 8 further exposed the performance of the model. These findings show how well the model detects trends in EEG data; although there is potential for development in lowering misclassifications, especially in terms of false positives and false negatives, overall the model performs really well.

**Figure 9:**
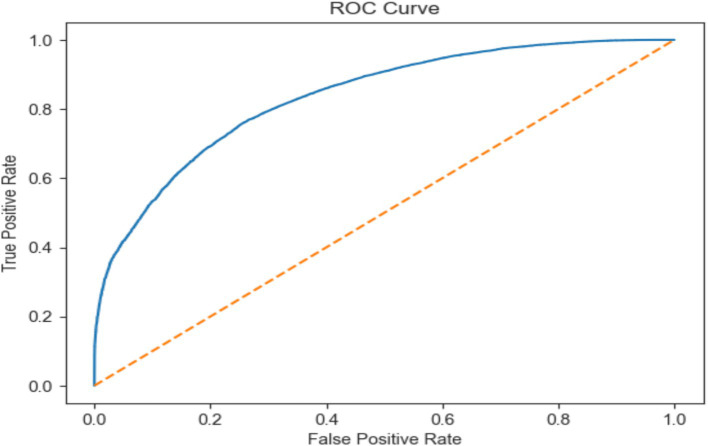
ROC AUC score of EEG data using the GB model.

These results demonstrate the potential of the GB model in accurately classifying epileptic and non-epileptic patients while also highlighting areas for further improvement through feature engineering, hyperparameter tuning, or ensemble methods, as shown in Figure 9.

### 4.4 Results of the RF model

**Figure 10:**
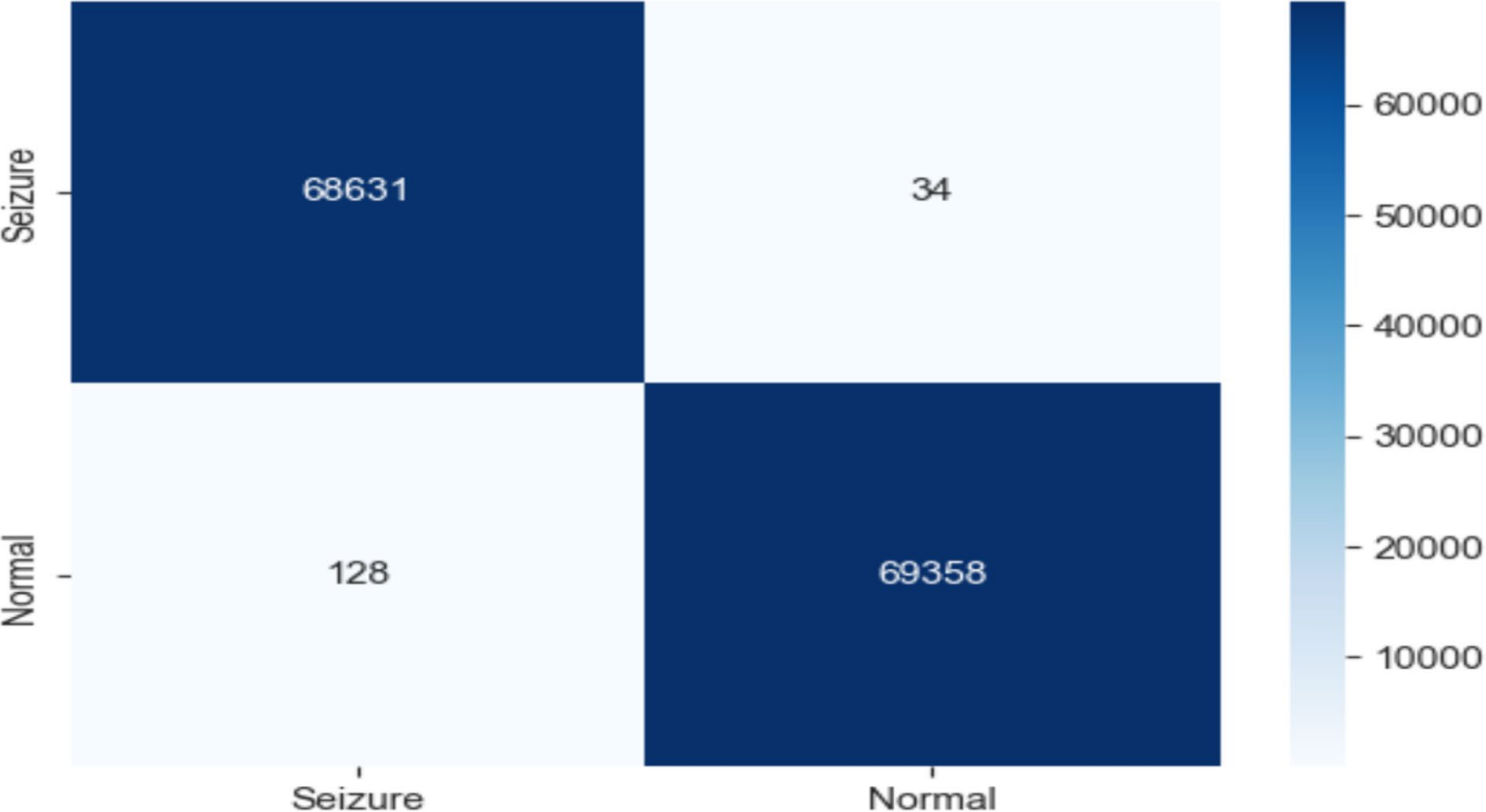
Confusion matrix of EEG data using the RF model.

As shown in Figure 10, the RF model was used with EEG traits to divide people into epileptic and non-epileptic groups. With an accuracy of 0.999, a precision of 1.000, a recall of 0.998, an F1 score of 0.991, and an ROC score of 1.000, the RF model showed extraordinary performance.

**Figure 11:**
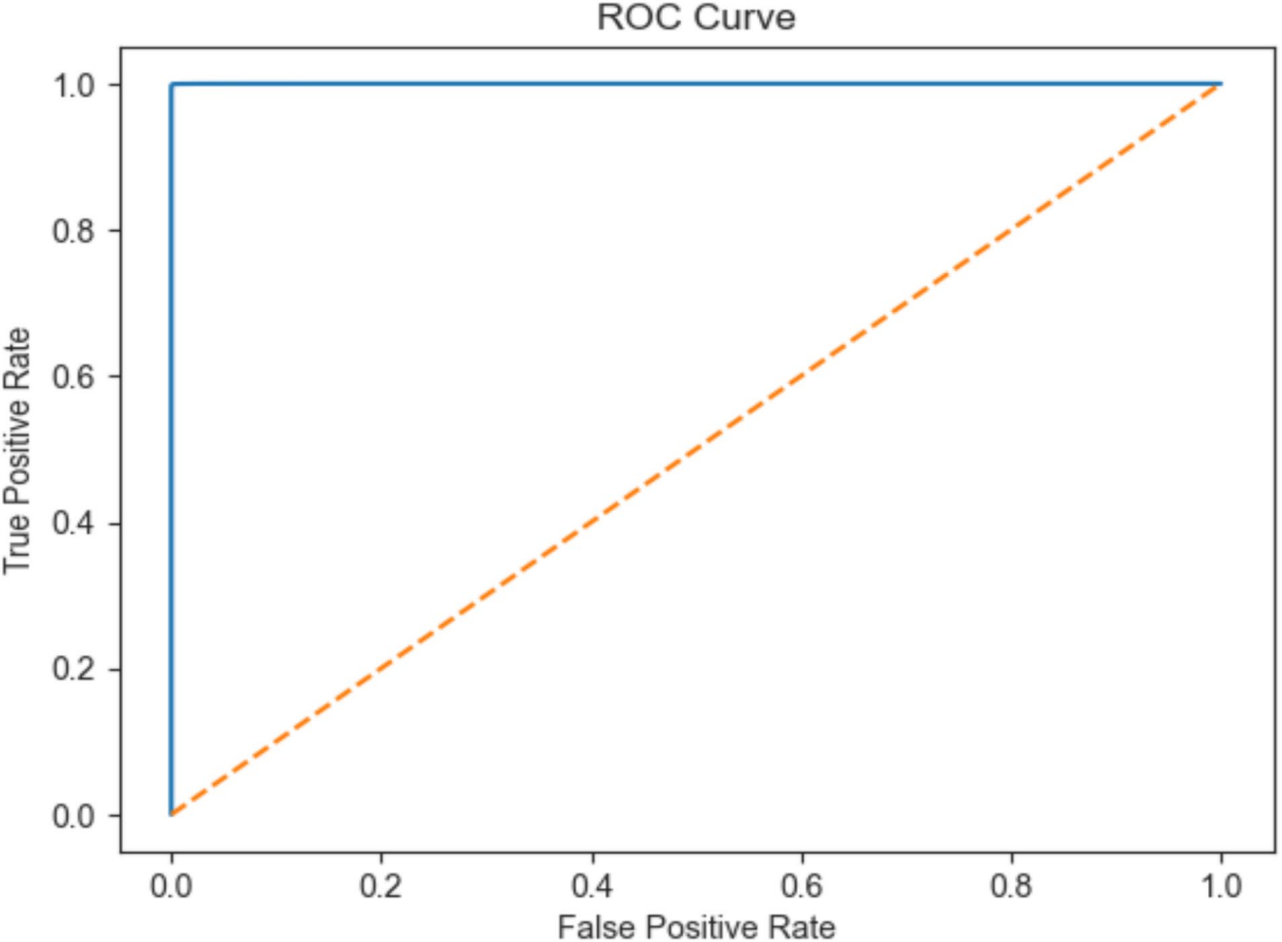
ROC AUC score of EEG data using the RF model.

The confusion matrix revealed 68,631 true negatives, 69,358 true positives, 34 false positives, and 128 false negatives. These exceptional results demonstrate the efficacy of the RF model in accurately classifying epileptic and non-epileptic patients based on the extracted EEG features, although further validation on independent datasets may be necessary to ensure generalizability, as shown in Figure 11.

### 4.5 Results of the K-NN

Normal from epileptic EEG data were distinguished using a K-NN classifier. Assigning the class of a data point depending on the majority class of its “k” closest neighbors in the feature space, K-NN is a basic, non-parametric classification method. This work selected K-NN with (𝑘 = 5), therefore classifying every EEG sample according on the majority vote of its five closest neighbors in the feature space.

**Figure 12:**
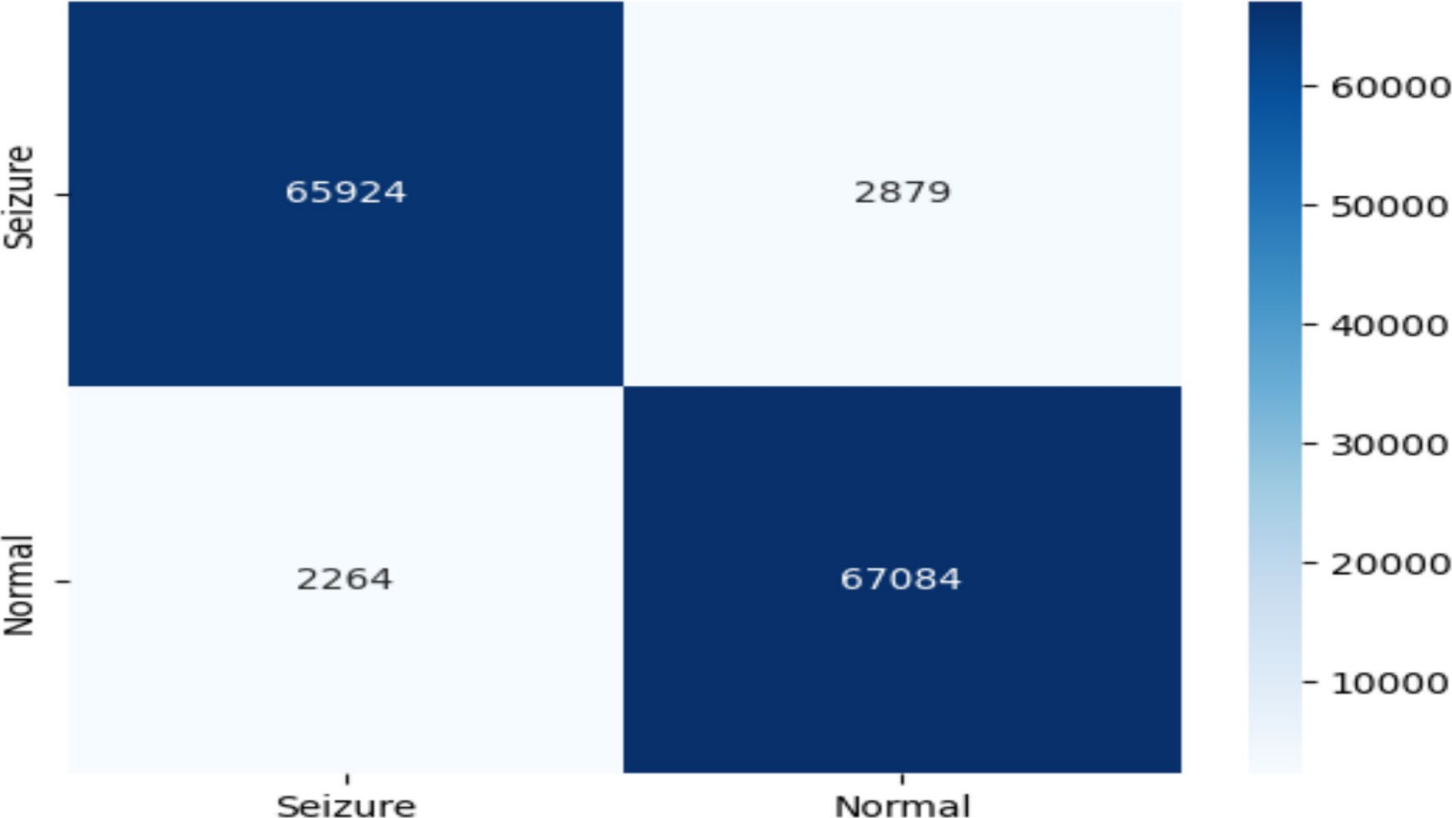
Confusion matrix of EEG data using the K-NN model.

The confusion matrix revealed that, out of the total predictions, 65,924 were true negatives and 67,084 were true positives, indicating that the majority of the normal and seizure cases were correctly identified. However, there were also 2,879 false positives and 2,264 false negatives, as shown in Figure 12.

**Figure 13:**
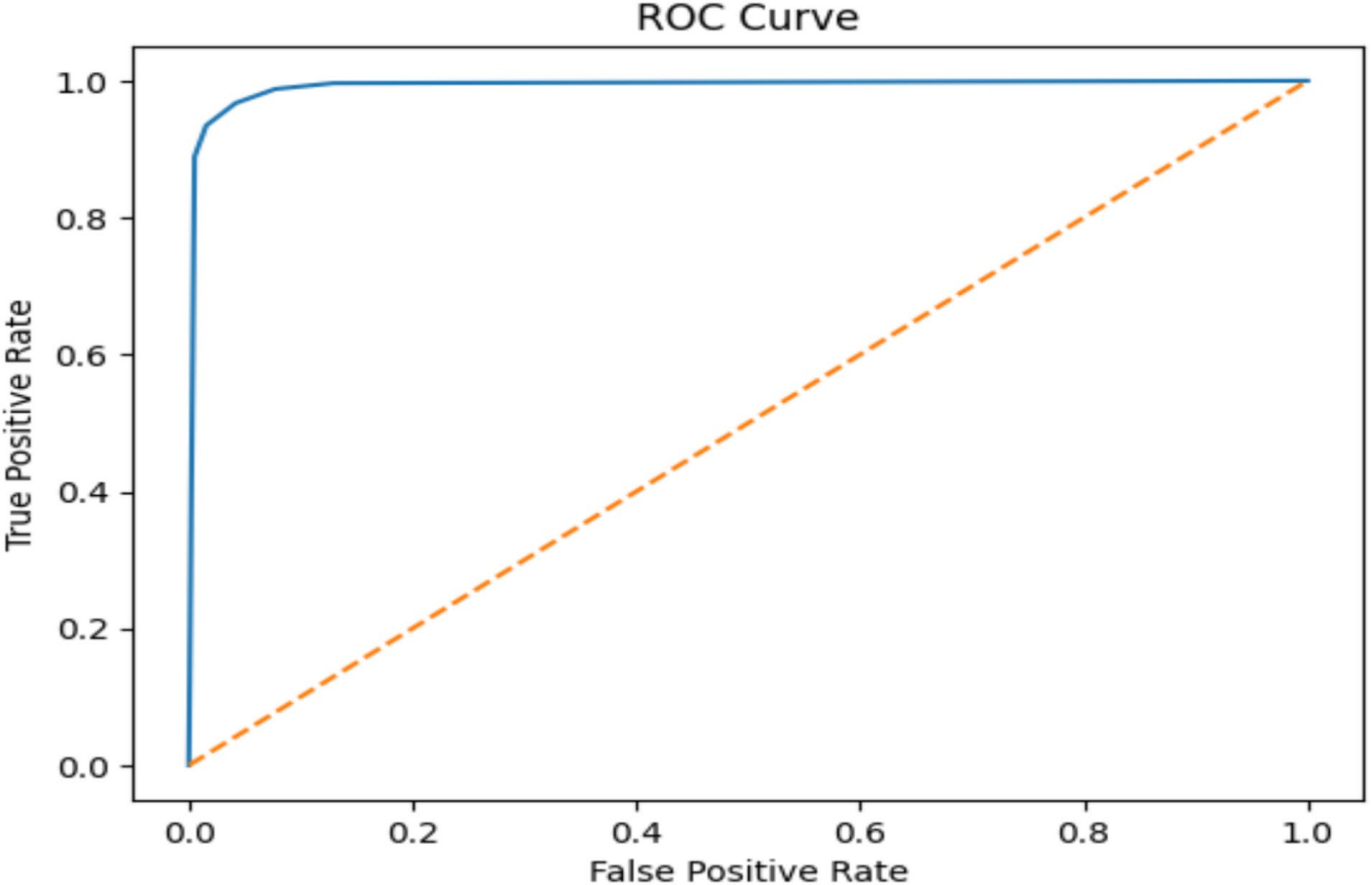
ROC AUC score of EEG data using the K-NN model.

The KNN model was shown scored with high accuracy (96.3%) indicates that the model correctly classified a substantial majority of the EEG signals. According the precision metric the KNN achieved 95.9% suggests that the model has a low rate of false positives, while recall of 96.7% indicates a low rate of false negatives. The ROC score of 99.02% further validates the model’s excellent ability to distinguish between normal and seizure cases, as illustrated in Figure 13.

These results show that although the model is highly accurate, there are still instances of misclassification, which is an area for potential improvement.

### 4.6 Results of the LSTM model

**Figure 14:**
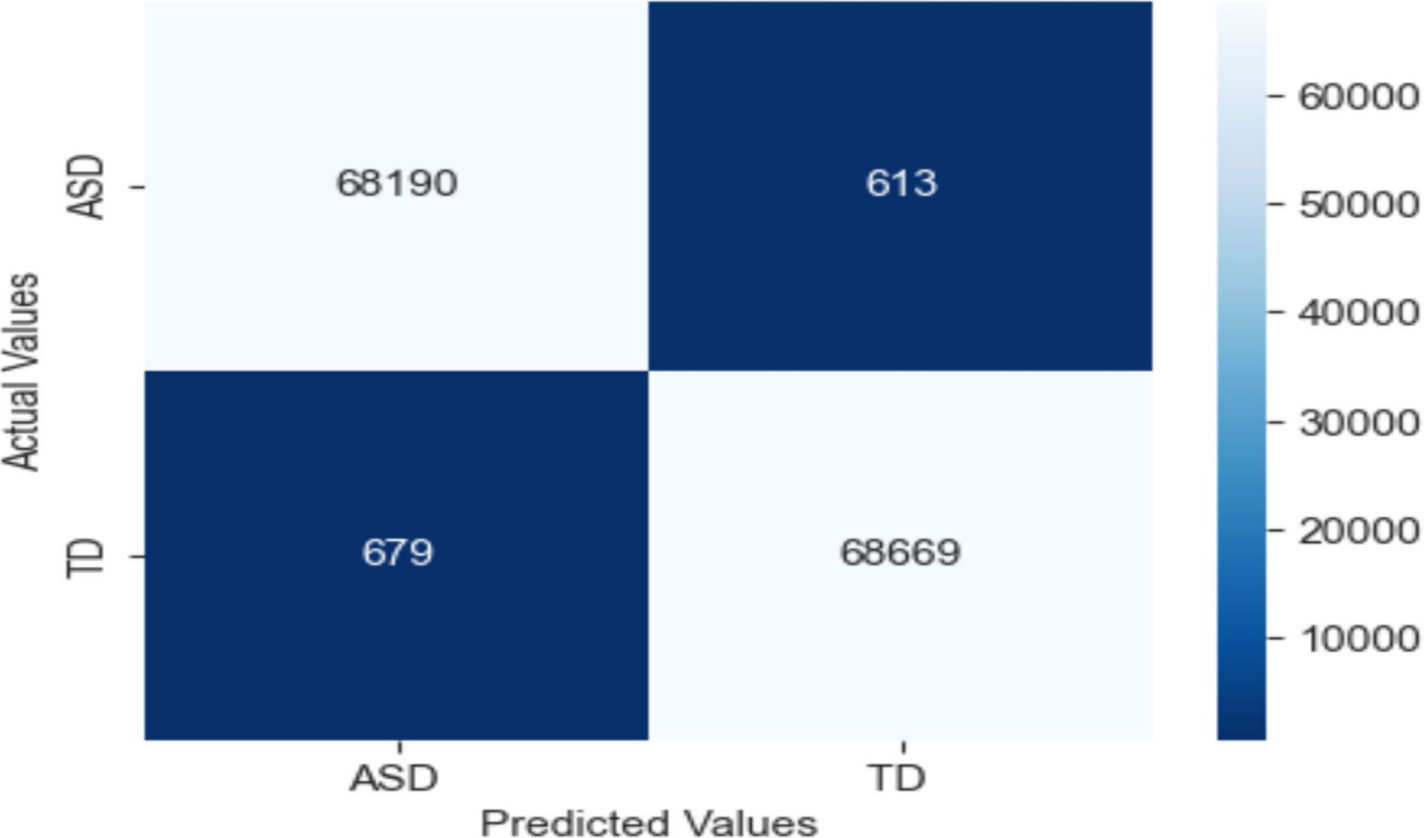
Confusion matrix of EEG data using the RF model.

In this experiment, classified epileptic and non-epileptic patients based on EEG signal characteristics using an LSTM neural network model. The LSTM model turned out with a 0.9906 accuracy. Table 6 gives the LSTM model’s parameters. As shown in Figure 14 the confusion matrix indicated 68,190 true positives, 613 erroneous positives, 68,669 true negatives, and 679 false negatives.

**Table 6:** LSTM model parameters using EEG data.

Parameter	Details
LSTM Layer	1,024
LSTM Layer	512, (BatchNormalization ())
LSTM Layer	256
Dense Lyer	34
Dense Lyer	1
Activation Function (Output Layer)	sigmoid
Optimizer	RMSprop
Learning Rate	0.001
Callback	EarlyStopping
Patience for No Improvement (EarlyStopping)	5 epochs
Epoch Training Stopped At	67 epochs
Maximum Epochs	150 epochs
Batch Size	1,024

**Figure 15:**
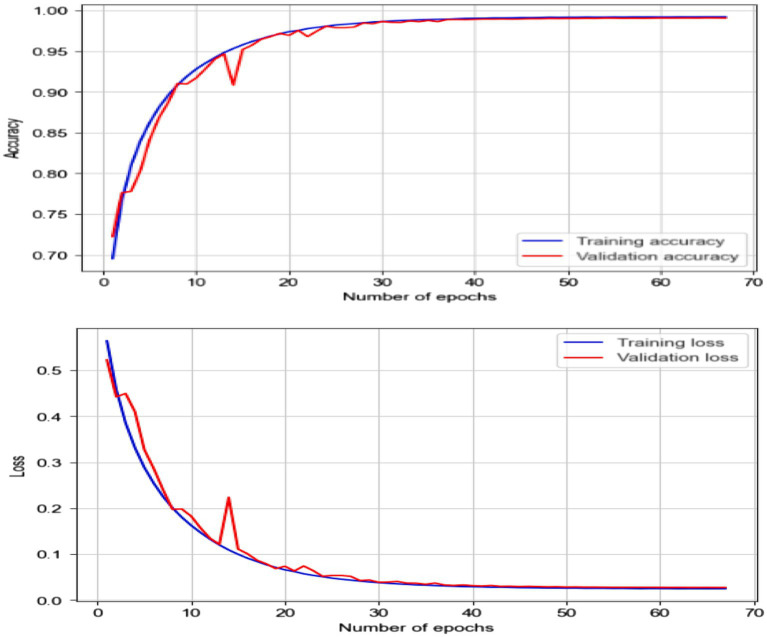
Accuracy and loss of EEG data using the LSTM model.

Using collected EEG data, the LSTM model showed remarkable accuracy of 99.06%, a precision of 99.12%, a recall of 99.02% and an F1 score of 99.07% for both epileptic and non-epileptic individuals. These findings demonstrate the great capacity of the model for precisely differentiating between the two classes, therefore stressing its possible uses in EEG-based diagnosis systems. Nevertheless, as Figure 15 shows, the LSTM was optimized and testing across EEG datasets and was shown the improvement in the generalizability of the model and guarantee its resilience in practical conditions.

### 4.7 Results of the LRCN model

Based on the features of the EEG data, this work categorized people as either epileptic or non-epileptic using an LRCN model. The LRCN model’s findings show that the accuracy was 0.9906; the precision was 0.9912; the recall was 0.9902; the F1 score was 0.9907. LRCN model characteristics and values (see Table 7).

**Table 7:** LRCN model parameters using EEG data.

Parameter	Details
ConvD1	filters = 64, kernel = 3, activation = ‘relu’
Custom Layer	Max Pooling
LSTM Lyer	1,024
LSTM Layer	512
LSTM	128
Dense Lyer	1
Activation Function (Output Layer)	sigmoid
Optimizer	RMSprop
Learning Rate	0.001
Callback	EarlyStopping
Patience for No Improvement (EarlyStopping)	5 epochs
Epoch Training Stopped At	69 epochs
Maximum Epochs	150 epochs
Batch Size	128

**Figure 16:**
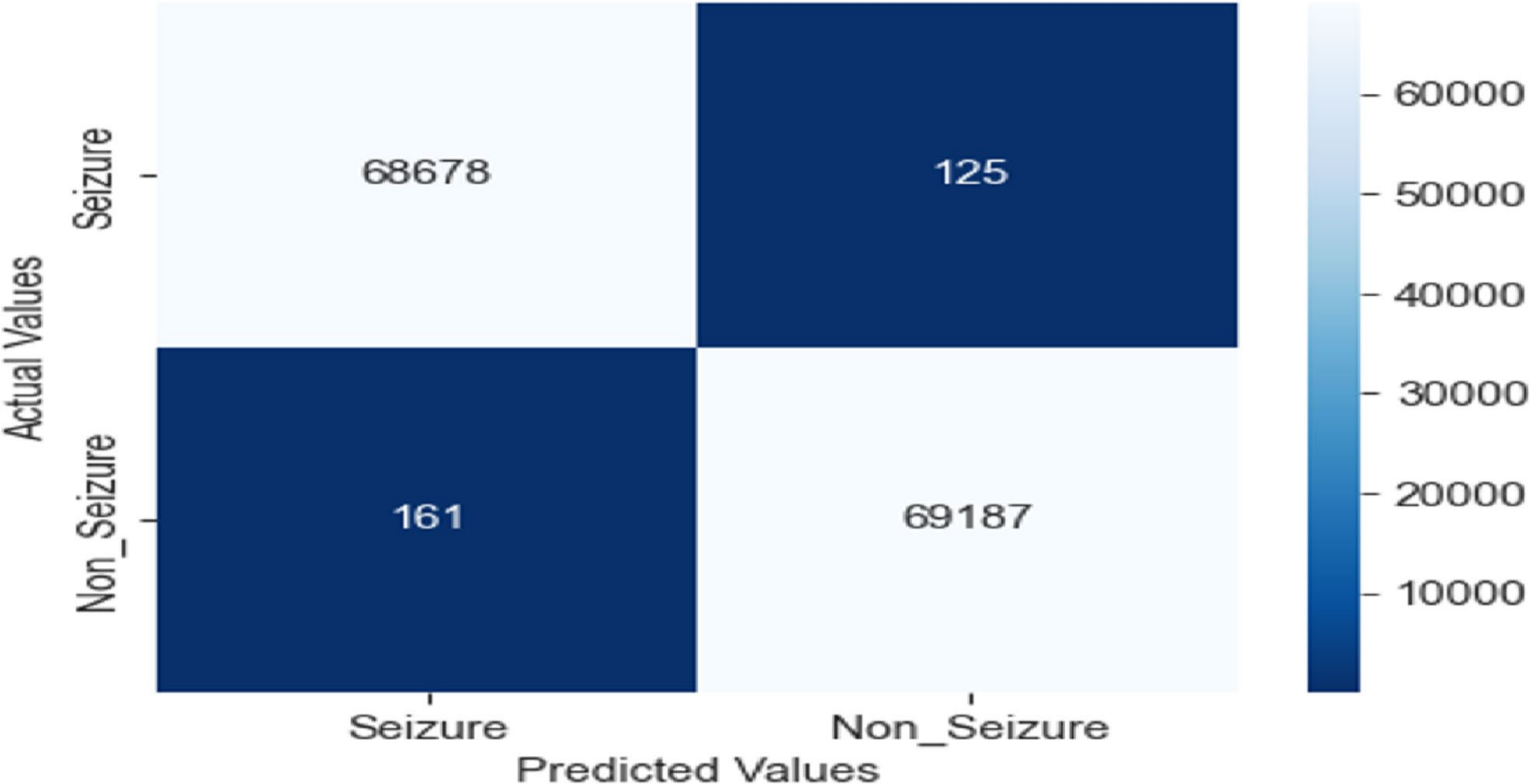
Confusion matrix of EEG data using the LRCN model.

Strong performance in categorizing seizure and non-seizure episodes from EEG data reveals in the confusion matrix for the LRCN model. The model fairly identifies most instances with 68,678 TP and 69,187 TN. Whereas (FP = 161) reveal minor misclassification of non-seizure events, false negatives (FN = 125) indicate a limited proportion of missed seizures. With low error, the high TP and TN values indicate outstanding sensitivity and accuracy, so the model is very dependable for monitoring epilepsy (see Figure 16).

**Figure 17:**
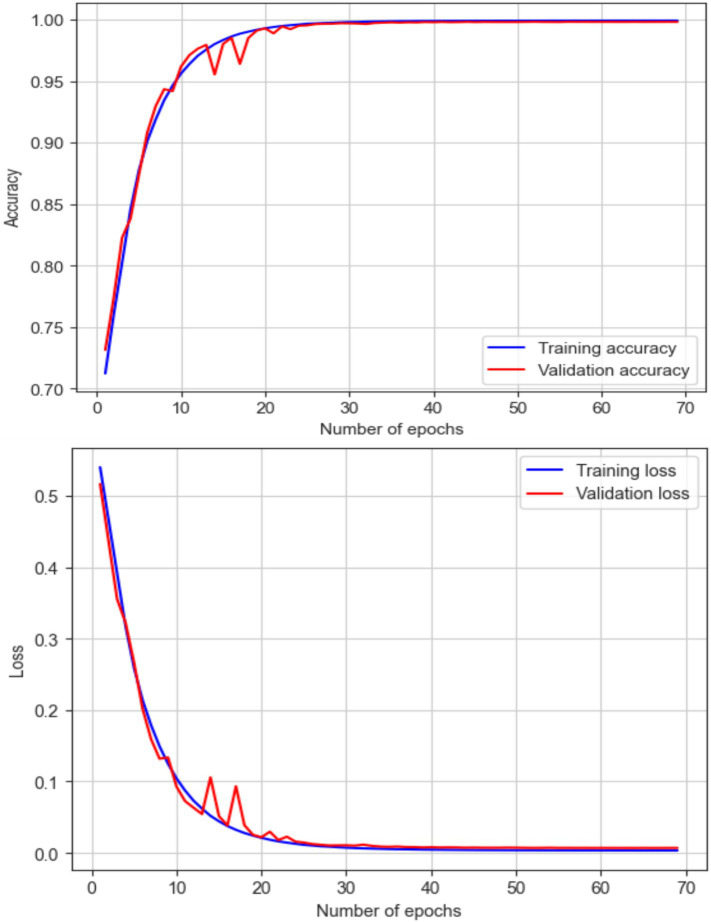
Accuracy and loss of EEG data using the LRCN model.

These results demonstrate the potential of the LRCN model in accurately classifying epileptic and non-epileptic patients based on the extracted EEG features, although further optimization and generalizability testing may be required, as shown in Figure 17.

### 4.8 Summary of the experimental results of the EEG classification

**Figure 18:**
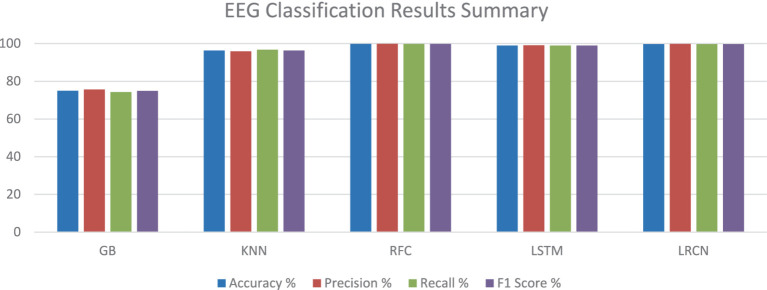
EEG classification results summary.

With almost perfect accuracy, precision, recall, and F1 score, the RF model exceeded the other models based on the testing findings in Section 4.3. Closely matching the RF model, the deep learning models, LSTM and LRCN, also showed outstanding performance with using various evaluation metrics. Though it performed really well, the GB model had somewhat worse measures than the other versions. With regard to reliably categorizing epileptic and non-epileptic patients based on EEG signal characteristics, the RF, LSTM, and LRCN models shown overall better performance; the RF model ranked highest in this regard in this research. Table 8 and Figure 18 help to show the outcomes.

**Table 8:** EEG classification results summary.

Model	Accuracy %	Precision %	Recall %	F1 score %
GB	75.0	75.6	74.3	74.9
KNN	96.3	95.9	96.7	96.3
RFC	99.8	99.9	99.8	99.8
LSTM	99.0	99.1	99.0	99.0
LRCN	99.7	99.8	99.7	99.7

### 4.9 EEG monitoring for detecting seizure behavior comparative

With a variety of techniques producing encouraging results, the subject of seizure detection and classification based on EEG data has experienced major developments recently. This review of 23 studies, along with our own research, highlights the diversity of techniques being applied to this critical medical challenge.

Traditional machine learning approaches continue to demonstrate their effectiveness, particularly when combined with innovative feature extraction methods. For instance, Rani and Chellam (8) achieved 99.60% accuracy using their Peak Signal Features method with an SVM classifier on the Bonn University dataset. Similarly, Almustafa (9) achieved 97.08% accuracy using a Random Forest classifier. These results underscore the continued relevance of classical machine learning techniques when applied with careful feature engineering.

Deep learning methods have shown remarkable performance in automatically learning relevant features from raw EEG data. Liu et al. (10) achieved a 97.4% F1-score using a hybrid bilinear deep learning network on the Temple University Hospital dataset, while Zhao et al. (11) reached 99.30% accuracy with a Linear Graph Convolution Network on the CHB-MIT dataset. These results demonstrate the power of deep learning in capturing complex patterns in EEG signals without the need for extensive feature engineering.

Hybrid and novel approaches have also yielded impressive results. Brari and Belghith (17) achieved 100% accuracy on the Bonn University dataset using a framework leveraging chaos and fractal theories. Kantipudi et al. (19) reported 99.6% detection performance with their complex model integrating wavelet-based filtering, bio-inspired optimization, and a specialized neural network. These innovative approaches show the potential for pushing the boundaries of seizure detection performance.

Our study, which achieved 99.9% accuracy using a Random Forest Classifier on a standard online dataset, aligns with and even surpasses many of the high-performing methods in the literature. This result underscores the potential of ensemble methods like Random Forest when applied to well-preprocessed EEG data.

The variability in datasets used across studies presents a challenge in directly comparing results. While some datasets like CHB-MIT and Bonn University are frequently used, allowing for some comparison, differences in preprocessing, feature extraction, and evaluation metrics can still make direct comparisons difficult. This highlights the need for standardized benchmarks and evaluation protocols in the field.

It’s noteworthy that while many studies report very high accuracies (>99%), real-world performance may differ due to factors such as inter-patient variability, noise in clinical settings, and the challenge of detecting seizure onset rather than ongoing seizure activity. Future research should focus on validating these high-performing models in diverse clinical settings and on larger patient populations.

The trend towards multimodal approaches, as seen in Hamlin et al. (26), and privacy-preserving methods, as in Ein Shoka et al. (20), points to future directions for the field. Integrating data from multiple sensor types and ensuring patient privacy will be crucial for the widespread adoption of automated seizure detection systems in clinical practice.

While the study demonstrates high accuracy (99.9%) in seizure detection, translating these models to wearable devices faces critical hurdles. Computational efficiency demands significant processing power, conflicting with the resource constraints of wearables. Real-time implementation requires low-latency pipelines, necessitating streamlined preprocessing and hardware-accelerated signal processing. Power consumption, patient-specific variability, and ambulatory noise (e.g., motion artifacts) further complicate reliability. Regulatory compliance, cost barriers, and the need for fail-safe mechanisms to minimize false alarms add layers of complexity. Addressing these challenges hinges on hardware sensor systems to balance accuracy with practicality for clinical adoption.

In conclusion, while significant progress has been made in seizure detection and classification, with our study contributing to the high-performance benchmarks, there remains room for improvement in areas such as real-time detection, generalizability across patients, and interpretability of complex models. Future work should focus on these challenges to bridge the gap between research performance and clinical applicability (see Table 9).

**Table 9:** EEG monitoring for detecting seizure behavior comparative.

Study	Model	Results
Our study	Random forest classifier	99.9% accuracy
Liu et al. (10)	Hybrid bilinear deep learning network	97.4% F1-score (TUH), 97.2% F1-score (EPILEPSIAE)
Fergus et al. (6)	k-NN classifier	88% sensitivity and specificity
Raghu et al. (7)	SVM with SDI feature	95.80–97.53% sensitivity, 0.4–0.57/h false detection rate
Rani and Chellam (8)	SVM with Peak Signal Features	99.60% accuracy
Almustafa (9)	Random Forest	97.08% accuracy
Zhao et al. (11)	Linear Graph Convolution Network	99.30% accuracy
Gabeff et al. (12)	CNN	0.873 F1-score, 90% seizure detection
Chou et al. (13)	CNN (various architectures)	97.7% accuracy (best model)
Kunekar et al. (15)	LSTM	97% validation accuracy
Mert and Akan (3)	Novel EEG analysis methodologies	97.89% accuracy
Brari and Belghith (17)	Chaos and fractal theory-based ML	100% accuracy
Shah et al. (18)	Random Neural Networks with DWT	93.27% (CHB-MIT), 99.84% (Bonn) accuracy
Kantipudi et al. (19)	FLHF, GBSO, and TAENN	99.6% detection performance
Zeng et al. (21)	Hybrid deep and shallow learning	Nearly 100% accuracy
Polat and Nour (24)	SVM with various kernels	76.70–82.50% accuracy
Hamlin et al. (26)	LDA with non-cerebral sensors	96% mean ROC value

To contextualize the performance of our proposed framework, we provide a detailed comparison with recent state-of-the-art methods in EEG-based seizure detection. Table 10 summarizes key metrics, datasets, and methodologies, emphasizing the strengths of our approach.

**Table 10:** Comparative analysis with state-of-the-art seizure detection approaches.

Study	Model/approach	Dataset	Accuracy	Sensitivity	Specificity	F1-Score
Our study	Random Forest (RF)	102 patients	99.9%	99.8%	99.9%	99.8%
Liu et al. (10)	Hybrid Bilinear CNN + RNN	TUH, EPILEPSIAE	97.2	–	–	97.4%
Zhao et al. (11)	Linear Graph ConvNet (LGCN)	CHB-MIT	99.3%	99.4%	98.8%	–
Kantipudi et al. (19)	GBSO-TAENN (Bio-inspired NN)	Undisclosed	99.6%	–	–	99.0%
Gabeff et al. (12)	CNN	REPO2MSE	90%	–	–	87.3%

Deploying EEG-based seizure detection in clinical settings faces computational and practical hurdles. While our Random Forest (RF) model achieves 99.9% accuracy with low latency (<10 ms) on CPUs, deep learning (DL) models like LSTM/LRCN require GPUs and exhibit higher latency (80–120 ms), limiting real-time use in wearables. Scalability and power constraints further favor RF, which processes 100 + EEG streams efficiently (~2 W) compared to DL’s GPU-dependent demands (~150 W). Additionally, long-term EEG monitoring poses comfort challenges, as patients must wear sensor caps for extended periods—a barrier for ambulatory use but manageable for admitted patients under supervision. For hospitalized individuals, continuous EEG provides critical insights despite discomfort, enabling timely interventions. Future work must address hardware miniaturization (e.g., flexible, wireless electrodes) and hybrid models to balance accuracy, comfort, and regulatory compliance (e.g., IEC 62304). These steps are vital to translate lab advancements into bedside solutions.

## 5 Conclusion

This study demonstrates that EEG signals remain a robust source for epileptic seizure detection, with the RF classifier achieving a remarkable 99.9% accuracy. Although deep learning models, such as LSTM and LRCN, also performed well, the superior results of RF underscore the relevance of traditional machine learning approaches in clinical seizure detection. These findings indicate that RF offers a viable solution for practical EEG-based seizure monitoring due to its accuracy and generalizability. However, the practical challenges associated with continuous, long-term EEG monitoring necessitate further exploration of alternative non-invasive monitoring techniques. Future research should focus on reducing the number of electrodes required for EEG-based detection without compromising accuracy, investigate dry electrode technologies, and integrate EEG with other modalities, such as video and EMG, for more comprehensive seizure monitoring solutions. Moreover, addressing the challenges of real-time detection and generalizability across diverse patient populations remains paramount for the widespread clinical adoption of EEG-based seizure detection systems.

## Data Availability

Publicly available datasets were analyzed in this study. This data can be found at: https://datadryad.org/stash/dataset/doi:10.5061/dryad.xsj3tx99w.
